# Abstracts from ISSN Brazil: Brasília, Brazil. 19-20 November 2016

**DOI:** 10.1186/s12970-017-0164-0

**Published:** 2017-03-14

**Authors:** André Barroso Heibel, Pedro Henrique Lopes Perim, Bryan Saunders, André Barroso Heibel, Déborah Michelly Abrantes, Caíque Fagundes Rauber, Caio Eduardo Gonçalves Reis, Jeferson Oliveira Santana, Diana Madureira, Elias de França, Caroline Ayme Fernandes Yoshioka, Marco Aurélio Lamolha, Cesar Augustus Zocoler, Paulo Roberto Sousa e Silva, Erico Chagas Caperuto, Janaína Lavalli Goston, Bárbara Ferreira dos Santos, Francine Rafaela Fernandes daSilva, Lívia Muniz Cirino de Carvalho, Sabrina Alves Ramos, Diana Madureira, Jeferson O. Santana, Elias de França, Leandro Gonçalves, Ronaldo V. T. Santos, Iris Callado Sanches, Carla C. Ramos, e Érico Chagas Caperuto, Caroline Romeiro, Gabrielle Goncalves Pires, Vitória C. A. R. A. Correia, Maria Cecilia F. Macedo, Octavio L. Franco, Breno Martins, Antonio Felipe Correa Marangon, Hugo Paulista, Pablo Almeida Macedo Norte, Danielle Almeida Da Fonseca, Debora Teixeira De Souza, Ana Gabriella Pereira Alves, Lana Pacheco Franco, Maria Sebastiana Silva, Pedro Henrique Lopes Perim, André Barroso Heibel, Bruno Gualano, Bryan Saunders, Elias de França, Diana Madureira, Yanesko Bella, Fabio Santos Lira, Jeferson O. Santana, Andre Fukushima, Alex Burton, Erico Chagas Caperuto, Artur P. de Azevedo, Lorruama J. Fogaça, Silvia L. Santos, João F. Mota, Gustavo D. Pimentel, Nayra Figueiredo, Marcela Queiroz, Jéssica Santos, João F. Mota, Gustavo D. Pimentel, Daniela S. Inoue, Valéria L. G. Panissa, Paula Alves Monteiro, José Gerosa-Neto, Fabrício E. Rossi, Erico C. Caperuto, Jason M. Cholewa, Alessandro M. Zagatto, Fábio S. Lira, Laís Monteiro Rodrigues Loureiro, Caio Eduardo Gonçalves Reis

**Affiliations:** 1 Laboratory of Nutritional Biochemistry, University of Brasilia, Brasilia, Federal District 70910-900 Brazil; 2 Applied Physiology & Nutrition Research Group, University of São Paulo, São Paulo, São Paulo 05508-900 Brazil; 3 São Camilo University Centre, São Paulo, São Paulo 04263-200 Brazil; 4 Laboratory of Nutritional Biochemistry, University of Brasilia, Brasilia, Federal District 70910-900 Brazil; 5 Catholic University of Brasilia, Brasilia, Federal District 71966-700 Brazil; 6 University Center of Brasília, Brasilia, Federal District 70790-075 Brazil; 7 GEPAME – São Judas Tadeu University, São Paulo, São Paulo 03166-000 Brazil; 8 Federal University of Minas Gerais, Belo Horizinte, Minas Gerais 31270-901 Brazil; 9 Catolic University of Minas Gerais, Belo Horizinte, Minas Gerais 30535-901 Brazil; 10 GEPAME – São Judas Tadeu University, São Paulo, São Paulo 03166-000 Brazil; 11 Federal University of São Paulo, São Paulo, São Paulo 04021-001 Brazil; 12 Program of Post-graduation in Physical Education, Catholic University of Brasilia, Brasilia, Federal District 71966-700 Brazil; 13 Graduation in Nutrition, Catholic University of Brasilia, Brasilia, Federal District 71966-700 Brazil; 14 Centre of Proteomic and Biochemical Analysis, Postgraduate Program in Biotechnology and Genomic Sciences, Catholic University of Brasilia, Brasilia, Federal District 71966-700 Brazil; 15 S-Inova Biotech, Graduate Program in Biotechnology, Dom Bosco Catholic University, Campo Grande, Mato Grosso do Sul 79117-900 Brazil; 16 Sports Nutrition Clinic Opeum, Brasilia, District Federal 70200-700 Brazil; 17 Natural Drugstore REDEPHARMA, Campina Grande, Paraiba 58400-085 Brazil; 18 Laboratory of Physiology, Nutrition and Health (LAFINS), Faculty of Physical Education and Dance (FEFD), Federal University of Goiás (UFG), Goiania, Goias 74690-900 Brazil; 19 Applied Physiology & Nutrition Research Group, University of São Paulo, São Paulo, São Paulo 05508-900 Brazil; 20 São Camilo University Centre, São Paulo, São Paulo 04263-200 Brazil; 21 Laboratory of Nutritional Biochemistry, University of Brasilia, Brasilia, Federal District 70910-900 Brazil; 22 GEPAME – São Judas Tadeu University, São Paulo, São Paulo 03166-000 Brazil; 23 Exercise and Immunometabolism Research Group, Department of Physical Education, Universidade Estadual Paulista, Presidente Prudente, São Paulo, 19060-900 Brazil; 24 Clinical and Sports Nutrition Research Laboratory (Labince), Nutrition Faculty (FANUT), Federal University of Goias (UFG), Goiânia, Goias 74690-900 Brazil; 25 Clinical and Sports Nutrition Research Laboratory (Labince), Nutrition Faculty (FANUT), Federal University of Goias (UFG), Goiânia, Goias 74690-900 Brazil; 26 Exercise and Immunometabolism Research Group, Department of Physical Education, Universidade Estadual Paulista, Presidente Prudente, São Paulo, 19060-900 Brazil; 27 Department of Sport, School of Physical Education and Sport, University of São Paulo, São Paulo, São Paulo 05508-900 Brazil; 28 Center and Prescription Motor Activity Laboratory, Department of Physical Education, Universidade Estadual Paulista, Presidente Prudente, São Paulo, 19060-900 Brazil; 29 Human Movement Laboratory, Universidade São Judas Tadeu, São Paulo, São Paulo 03166-000 Brazil; 30 Department of Kinesiology, Coastal Carolina University, Conway, SC USA; 31 Department of Physical Education, of Universidade Estadual Paulista, Bauru, São Paulo 17033-360 Brazil; 32 Laboratory of Nutritional Biochemistry, University of Brasilia, Brasilia, Federal District 70910-900 Brazil

## P1 An update on extracellular buffering agents to improve exercise capacity and performance

### André Barroso Heibel^1^, Pedro Henrique Lopes Perim^2,3^, Bryan Saunders^2^

#### ^1^Laboratory of Nutritional Biochemistry, University of Brasilia, Brasilia, Federal District, 70910-900, Brazil; ^2^Applied Physiology & Nutrition Research Group, University of São Paulo, São Paulo, São Paulo, 05508-900, Brazil; ^3^São Camilo University Centre, São Paulo, São Paulo, 04263-200 Brazil

##### **Correspondence:** Bryan Saunders (drbryansaunders@outlook.com)

**Background**

Agents capable of increasing extracellular buffering capacity to combat exercise induced acidosis have been researched for the better part of a century. Sodium bicarbonate, sodium citrate and sodium/calcium lactate supplementation all result in increased circulating bicarbonate and have independently been shown to improve exercise capacity and performance. And yet evidence regarding the efficacy of these buffering agents is inconsistent and recent developments are providing novel information to suggest that current dosing strategies may be suboptimal for some individuals.

**Method**

Literature data were reviewed seeking articles published until October 2016 using the following terms: “buffering”, “sodium bicarbonate”, “sodium citrate” or “sodium/calcium lactate”. Experimental trials and review papers were selected to be analyzed. Studies and sample characteristics, biochemical markers, and performance parameters according to supplement intake were appraised.

**Results**

Sodium bicarbonate effects are well established, although high intra- and inter individual variability in the exercise responses to supplementation exist and may be influenced by associated side-effects. A recent study showed that individuals differ substantially in the time at which they attain peak bicarbonate levels in blood, suggesting the need to individualise supplement timing. Sodium citrate appears to demonstrate an inefficacy to improve exercise capacity and performance. However, novel data suggests that previous research is limited by supplementation timing and side-effects, meaning further investigation is needed to establish its effects on exercise using a more optimal dosing strategy. Evidence to support the use of lactate supplementation as a buffering agent to improve exercise is scarce and contradictory. Research has shown large (+17%), moderate (+1.7%) or no improvements in exercise following supplementation. Emerging evidence from our laboratory suggest that chronic supplementation with calcium lactate does not increase blood buffering capacity or improve high-intensity exercise performance, casting doubt on its efficacy as an ergogenic aid.

**Conclusion**

Sodium bicarbonate’s ergogenic potential is clear, although the contributing factors to variability in responses warrants further investigation. The efficacy of sodium citrate and lactate supplementation to improve exercise is unclear and controversial; more well controlled investigations are necessary to elucidate their ability to improve buffering capacity and subsequent exercise. In conclusion, more research is required to determine how to optimise supplementation of all these buffering agents to increase the likelihood of an improved exercise capacity or performance

## P2 Does caffeine and carbohydrates co-ingestion improve endurance performance? – a systematic review 

### André Barroso Heibel^1^, Déborah Michelly Abrantes^2^, Caíque Fagundes Rauber^3^, Caio Eduardo Gonçalves Reis^1^

#### ^1^Laboratory of Nutritional Biochemistry, University of Brasilia, Brasilia, Federal District, 70910-900, Brazil; ^2^Catholic University of Brasilia, Brasilia, Federal District, 71966-700, Brazil; ^3^University Center of Brasília, Brasilia, Federal District, 70790-075, Brazil

##### **Correspondence:** André Barroso Heibel (andreheibel@gmail.com)

**Background**

Carbohydrate and caffeine has been used as ergogenic aid for endurance sports long since. However, the additional benefits after caffeine and carbohydrate coingestion on performance remain uncertain. Thus, the aim of this systematic review is to evaluate whether the intake of caffeine plus carbohydrate can improve endurance performance more than carbohydrate alone.

**Methods**

A systematic literature search was conducted on PubMed database (English, Spanish and Portuguese) seeking articles published until September 2016 using a combination of the following keywords: ‘caffeine’, ‘carbohydrate’, ‘endurance’, ‘exercise’, and ‘performance’. Clinical trials with human subjects which analyzed the effects of caffeine and carbohydrate co-ingestion on endurance exercise performance compared with carbohydrate were included. Studies and sample characteristics, biochemical markers, and performance parameters according to the supplement intake timing (‘before’, ‘during’, or ‘both’) were appraised.

**Results**

Thirty-four studies were screened, fifteen of them were excluded after the eligibility assessment and nineteen were selected for the final analysis. Compared with carbohydrate alone, the caffeine (1.8 to 3.0 mg/kg*h-1) and carbohydrate (0.68 to 0.84 mg/kg*h-1) co-ingestion made 3–4 times during a cycling exercise leads to an improvement of 3.3–4.4% on time trial and 27% on time to exhaustion protocols in highly trained male athletes (65.7–73.8 VO2max). Moreover, 5 mg/kg of caffeine plus 5 mL/kg of Gatorade® coingestion one hour before a cycling time to exhaustion test (80% VO2max) showed a ~20% amelioration on performance in male and female athletes (51–52 VO2max). Furthermore, caffeine (1.8 to 3 mg/kg*h-1) and carbohydrate (0.68 mg/kg*h-1) co-ingestion before and during (every 15 min) a task-specific soccer training seems to improve speed and vertical jump in male athletes (50–56 VO2max). Regarding others sports, a 2000 m rowing time trial and a Rugby shuttle test protocol can be benefited by caffeine and carbohydrate coingestion.

**Conclusion**

These findings suggest that caffeine and carbohydrate co-ingestion before and during a cycling and soccer can improve exercise’s performance. These results may be attributed to the adenosine’s inhibition and catecholamine’s (adrenalin and noradrenalin) stimulus and its consequent lipolytic effects. More studies need to explore the exactly mechanisms in that co-ingestion of caffeine and carbohydrate can improve athletic performance.

## P3 The influence of supplement acute inorganic nitrate in sprints of 400 meters

### Jeferson Oliveira Santana, Diana Madureira, Elias de França, Caroline Ayme Fernandes Yoshioka, Marco Aurélio Lamolha, Cesar Augustus Zocoler, Paulo Roberto Sousa e Silva, Erico Chagas Caperuto

#### GEPAME – São Judas Tadeu University, São Paulo, São Paulo, 03166-000, Brazil

##### **Correspondence:** Erico Chagas Caperuto (ecaperuto@yahoo.com)

**Background**

Nitrate is a known precursor of nitric oxide which has many benefits in high-intensity sports, due to its vasodilating effect, decrease in adenosine triphosphate cost and improvement in the physiological responses of fast twitch fibers (type II). Adherence to running have been increasing and performance optimization is being chased by runners. They used strategies such as ~400m sprints at the beginning and in the end of training and races.The aim of this study was to evaluate the influence of acute nitrate supplementation on performance markers in runners during repeated 400m sprints.

**Methods**

In a double blind crossover design 12 runners (aged 20-40 years) were randomly assigned to one of two conditions: ingestion of 750mg (two capsules) of inorganic nitrate and/or 750mg of resistant starch (placebo). In both conditions participants ingested the supplementation 3 hours (in a fasted state) before the 4x 400m sprint test (an all-out 4x 400m interspersed by 5 minutes between them). The variables assessed during the sprint test were: total time of each sprint and blood plasma lactate concentration [La]p PRE-SPRINT (before each sprint) and POST-SPRINT (after each sprint). After the normality test (Shapiro-Wilk) the two-way ANOVA (with Tukey post-test) was performed (with p <0.05).

**Results**

There was no group interaction (p >0.05) for total sprint time of each sprint. However, there was a significant effect of time for both conditions. We observed an increase in the total time to perform the 3^rd^ Sprint compared to 2^nd^ Sprint and to the 1^st^ Sprint (1^st^ Sprint 87.17 ± 10.25 < 3^rd^ Sprint 96.83 ± 24.28 > 2^nd^ Sprint 92.00 ± 18.79;) as well as for the 4^th^ sprint compared to 2^nd^ Sprint and the 1^st^ Sprint (1^st^ Sprint 87.17 ± 10.25 < 4^th^ Sprint 98.83 ± 18.68 > 2^nd^ Sprint 92.00 ± 18.79). In the Nitrate condition there was an increase in the total sprint time, only in the 4^th^ Sprint compared to 3^rd^ Sprint and 2^nd^ Sprint (2^nd^ Sprint 88.67 ± 15.62 < 4^th^ Sprint 94.17 ± 16.87 > 3^rd^ Sprint 90.83 ± 17.12). There was a group interaction for [La]p (p < 0.05). There were significant differences in [La]p POST-SPRINT at the 2^nd^ sprint (Placebo: 13.20 ± 5.36; Nitrate: 7.53 ± 3.08). There was a significant effect of time for [La]p for both conditions (p < 0.05). The Placebo condition showed a significant increase in [La]p POST-SPRINT compared to PRE-SPRINT in the 1^st^ Sprint (PRE-SPRINT 2.38 ± 1.05; POST-SPRINT 9.42 ± 4.04) in 2^nd^ Sprint (PRE-SPRINT 9.07 ± 6.40; POST-SPRINT 13.20 ± 5.36) and 3^rd^ Sprint (PRE-SPRINT 10.65 ± 4.20; POST-SPRINT 13.53 ± 6.48) with stable values only in the 4^th^ Sprint (PRE-SPRINT 13.40 ± 6.97; POST-SPRINT 13.20 ± 5.36). In the Nitrate condition there was a significant increase from PRE-SPRINT to POST-SPRINT only in 1^st^ Sprint (PRE-SPRINT 2.67 ± 0.76; POST-SPRINT 6.90 ± 2.20); in the 2^nd^ Sprint (PRE-SPRINT 6.50 ± 2.27; POST-SPRINT 7.53 ± 3.08), 3^rd^ Sprint (PRE-SPRINT 8.62 ± 3.05; POST-SPRINT 10.38 ± 3.02) and 4^th^ Sprint (PRE-SPRINT 13.40 ± 6.97; POST-SPRINT 10.30 ± 3.42) it did not change significantly.

**Conclusion**

Acute inorganic nitrate supplementation reduces blood lactate levels and may improve performance in sprints of 400 meters.

## P4 Preparation of pratical snacks destined for athletes and practitioners of physical activity

### Janaína Lavalli Goston^1^, Bárbara Ferreira dos Santos^2^, Francine Rafaela Fernandes da Silva^2^, Lívia Muniz Cirino de Carvalho^2^, Sabrina Alves Ramos^2^

#### ^1^Federal University of Minas Gerais, Belo Horizinte, Minas Gerais, 31270-901, Brazil; ^2^Catolic University of Minas Gerais. Belo Horizinte, Minas Gerais, 30535-901, Brazil

##### **Correspondence:** Janaína Lavalli Goston (janaina@janainagoston.com)

**Background**

Due the troubled routine experienced by most, people are failing to prepare your own meal. Concomitantly, there is an increase in the number of physically active people. Thus, this summary aims to report the development of healthy and practical snacks for this audience, with suitable sensory characteristics.

**Methods**

The study was conducted at the Pontifícia Universidade Católica de Minas Gerais (PUC Minas), campus Barreiro, during the first half of 2016. Original recipes were prepared by members of the project and adaptations in pre-disclosed recipes in cookbooks, or social internet networks. For each preparation were prepared Sheets Preparation Techniques (SPT), in order to standardize the recipes. In addition to concerns about the quality and nutritional value of the ingredients, the preparation has been done by optimal dietary techniques for obtaining tasty preparations and practices, that may contribute to improved physical performance and health promotion who consume them.

**Results**

Altogether were prepared 78 recipes, been 45 savory preparations, 11 drinks and 22 sweet preparations. Of total revenues, 46 were classified as easy to transport because of the texture of the preparation, size and moisture content, which can preserve their sensory characteristics, to facilitate packaging for consumption at locations outside the home and contribute to maintaining the hygienic quality health. The recipes were classified by the time of intake to activity ("pre or post workout"), providing energy and nutrients, especially the available sources of carbohydrates and proteins. Of 78 revenues prepared, 25 were classified exclusively as pre-training, 35 were classified exclusively as a post workout and 7 were classified as pre and post-training, having good functionality for both occasions. Before training predominated preparations with low carbohydrate sources to moderate glycemic index, low in fiber and fat to facilitate gastric emptying and moderate in protein. After training, they predominated meals sources of moderate carbohydrate high glycemic index, fundamental to the replenishment of muscle glycogen stores, protein offered in minimum amount of 10g to promote protein synthesis and muscle recovery. With the use of these revenues, to facilitate access and preparation of healthy foods, it is possible to value the act of cooking based on the principles of gastronomy, collude with the nutritional principles addressed in the current Guia Alimentar para População Brasileira for healthy eating, as well as ensuring the premises of Sports Nutrition whose nutritional strategies can promote the improvement of physical performance.

**Conclusion**

The research helped to expand studies on the development of new recipes and food combinations that will foster innovation, diversity, health promotion, improved palatability and physical performance, while optimizing time, since these revenues are practical, easy to transport and, not least, tasty, with a view to run day-to-day that most people have and the importance of pleasure in food quality.

## P5 Comparison between palatinose, dextrose and waxy maize glycemix curve, at rest and during exercise

### Diana Madureira^1^, Jeferson O. Santana^1^, Elias de França^1^, Leandro Gonçalves^1^, Ronaldo V.T. Santos^2^, Iris Callado Sanches^1^, Carla C. Ramos^1^ e Érico Chagas Caperuto^1^

#### ^1^GEPAME – São Judas Tadeu University, São Paulo, São Paulo, 03166-000, Brazil; ^2^Federal University of São Paulo, São Paulo, São Paulo, 04021-001, Brazil

##### **Correspondence:** Erico Chagas Caperuto (ecaperuto@yahoo.com)

**Background**

Glycemic index is how fast carbohydrates appear in the blood. Different kinds of carbohydrates affect blood glucose unequally, both at rest and in exercise. The aim of this study was to compare the effects of palatinose, dextrose and waxy maize ingestion on blood glucose, in two different situations, at rest and in exercise.

**Methods**

7 adults, both genders, took part in the study in six different days, three for each situation (rest and exercise). After three hours of fasted state they were randomly assigned to drink 25g of palatinose, dextrose or waxy maize, diluted in 300ml of water. Blood glucose was measured before the beverage consumption and in the next 60 minutes every 15 minutes, for both situations. Immediately after the supplement consumption, all subjects performed a one hour run in a treadmill, at 70% of the estimated maximum heart rate.

**Results**

Both palatinose and waxy maize promoted a mild increase in blood glucose levels in the first 15 minutes (0min:82.9 ± 11.7; 15min:103.3 ± 15.2; 0min:84.2 ± 11.6; 15min:95.8 ± 14.3), and remained high up to 60 minutes. Dextrose showed a higher increase in blood glucose at 15 and 30 minutes when compared to the other substances (Dextrose – 0min:83.3 ± 5.6; 15min:123.0 ± 18.6; 30min:123.1 ± 20.3; Palatinose - 0min: 82.9 ± 11.7; 15min:103.3 ± 15.2; 30min: 103.4 ± 11.2; Waxy Maize – 0min: 84.2 ± 11.6; 15min: 95.8 ± 14.3; 30min: 96.4 ± 9), than, at 60 minutes, blood glucose returned to its initial values. At exercise, waxy maize glucose levels showed no differences during the test (0min: 87.3 ± 4.9). Dextrose showed a not significant increase at 15 and 30 minutes, and returned to its initial values right after (0min:86.3 ± 11.2; 15min: 96 ± 15.6; 30min: 93.3 ± 14.4). Palatinose showed blood glucose reduction at 15 minutes, remaining that way during the rest of the test (0min:95.8 ± 7.4; 15min:80.5 ± 9.7). Blood glucose level at 15 minutes was higher with dextrose when compared to the other substances (Dextrose:96.0 ± 15.6; Palatinose:80.5 ± 9.7; Waxy Maize:80.8 ± 10.0). After 15 minutes of exercise, blood glucose increased with Dextrose consumption, meanwhile with the other tested substances it was reduced.

**Conclusion**

There is a unique relationship between the type of carbohydrate, its molecule organization, glycemic index and how it is processed by the organism, at rest and during exercise.

## P6 Glycemic evaluation during incremental test associated with fructooligosaccharides on diet

### Caroline Romeiro^1^, Gabrielle Goncalves Pires^2^, Vitória C.A.R.A. Correia^2^, Maria Cecilia F. Macedo^2^, Octavio L. Franco^3,4^

#### ^1^Program of Post-graduation in Physical Education, Catholic University of Brasilia, Brasilia, Federal District, 71966-700, Brazil; ^2^Graduation in Nutrition, Catholic University of Brasilia, Brasilia, Federal District, 71966-700, Brazil; ^3^Centre of Proteomic and Biochemical Analysis. Postgraduate Program in Biotechnology and Genomic Sciences, Catholic University of Brasilia, Brasilia, Federal District, 71966-700, Brazil; ^4^S-Inova Biotech, Graduate Program in Biotechnology, Dom Bosco Catholic University, Campo Grande , Mato Grosso do Sul, 79117-900, Brazil

##### **Correspondence:** Caroline Romeiro (ntr.carolineromeiro@gmail.com)

**Background**

The gut microbiota is known to play a key role in host metabolism. One of the major bacterial fermentation products of non-digestible carbohydrates are the short chain fatty acids (SCFAs) including acetate, butyrate and propionate. The SCFAs can be used by the gut epithelial cells as energy source and utilized as energetic substrate in other tissues such as muscle and liver. The beneficial moderate exercise effects on gut physiology and microbiota are well-known, but not the related prolonged exercise effects. Prolonged exercise such as endurance training could associated to colon ischemia altering the gut permeability that can decrease exercise performance. Therefore, this study aims to examine the glycemic response in a mice incremental test when fructooligosaccharides (FOS) on diet and endurance exercise are associated.

**Methods**

Thirty-two 8-week old C57Bl/6J male mice were randomly allocated in four groups: 1. normal diet and sedentary (NS); 2. FOS diet and sedentary (FS); 3. normal diet and exercise (NE); 4. FOS diet and exercise (FE). After 4 and 8 weeks, all animals performed incremental test and every 3 min test, the treadmill speed increased 3 m.min-1. A 5μl blood sample was collected from animals tail and the blood glucose was determined at each test stage. The exercise groups trained during 8 weeks on a treadmill 50% of maximal velocity (Vmax) for 60 min, 5 times a week. After 8 weeks training, the exercised groups were submitted to a test until exhaustion. All animals were euthanatized and duodenum and colon tissues were fixed for histology. Gut microbiota modulation will be analyzed by stool sample 16S gene pyrosequencing of.

**Results**

Initially 66.6% and 87.5% of the animals fed with FOS diet (FE and FS groups) showed higher blood glucose at incremental test end by at least 21.65% of the initial blood glucose. No animal in NE group presented the final blood glucose enhanced in comparison to initial blood glucose and only 25% in NS group showed similar glycemic behavior. The FOS group ran longer distances in incremental test at eighth week. Corresponding distances from each group were 347 m (±87.77 m) FE vs 279 m (± 39.44m) NE, and 331m (± 95.46 m) FS vs 227m (± 29.16m) NS.

**Conclusion**

The FOS diet seems to promote glycemic control and improve the distance running. Future results can elucidate whether the gut microbiota modulation can help in improving mice endurance exercise performance.

## P7 Effects of administration of zma and tribulus terrestris in body composition and on the hormone responses in adult women practitioners of resistance training

### Breno Martins, Antonio Felipe Correa Marangon, Hugo Paulista

#### Sports Nutrition Clinic Opeum – Brasilia, District Federal, 70200-700, Brazil

##### **Correspondence:** Antonio Felipe Correa Maragon (felipe@opeun.com.br)

**Background**

In search of muscle performance many individuals look beyond physical activity and nutrition, supplements that can contribute to this performance also improving body composition. So we see many fans of products marketed under this proposal, labeled "hormone optimizers," which are composed of mixtures of various ingredients that supposedly increase endogenous testosterone levels, which are known to increase muscle protein synthesis, resulting increases in muscle mass and strength. Therefore, the overall objective of this study was to evaluate the body composition and hormonal concentrations of total testosterone, free and luteinizing hormone (LH) after administration of 8 weeks of ZMA and Tribulus terrestris in young women resistance training practitioners (bodybuilding). The purpose of this study was to assess body composition and hormonal concentrations of total and free testosterone and LH after administration of 8 weeks of ZMA (zinc, magnesium and vitamin B6) and Tribulus terrestris (TT) in young women practicing resistance training (bodybuilding).

**Methods**

The study included 20 apparently healthy women and trained in resistance exercise. The volunteers used for eight weeks, different supplements were divided into four groups: 1) ZMA; 2) TT; 3) ZMA + TT; 4) Placebo Group (PG). At the beginning of the study (day 1), on the 29th day and the day after the end of supplementation (57 days), all volunteers underwent anthropometric measurements, which were collected, body weight, height and skinfold thickness, and were held blood collections in order to measure the hormones. To analyze the differences between groups and intra-group the Kruskal-Wallis test and ANOVA with the Friedman parameter were applied respectively. When it indicated the difference the correction procedure Bonferroni post hoc was used. A level α of P ≤ 0.05 was used as a statistical difference.

**Results**

ZMA + TT group showed higher LH (9.00 mIU / ml) compared to the placebo group (GP) (3.72 mIU / ml; P = 0.014) and higher value against the ZMA group (5.44 mIU / ml; P = 0.048). However, after Bonferroni correction compared to the difference ZMA group became zero. There were no significant differences in total and free testosterone between groups. In relation to body composition lean mass of the ZMA group increased significantly when 3 (48.16 kg) in relation to the time 1 (start of supplementation) (47.27 kg), but was not statistically significant between the groups (P < 0.05).

**Conclusion**

It is concluded that supplementation of ZMA and TT for eight weeks does not alter the hormonal concentrations of total and free testosterone, LH except when compared to the placebo group. In relation to body composition significant increase in lean body mass in the ZMA group, but no statistically significant differences between the other groups.

## P8 Orthorexia nervous: health excess that may cause disturbances

### Pablo Almeida Macedo Norte, Danielle Almeida Da Fonseca, Debora Teixeira De Souza

#### Natural Drugstore REDEPHARMA, Campina Grande, Paraiba 58400-085, Brazil

##### **Correspondence:** Pablo Almeida Macedo Norte (nutpablonorte@gmail.com)

**Background**

Eating Disorders (DE) are severe psychiatric disorders considered important health problems. A recently introduced framework is called Orthorexia Nervous. Exposed as a pathological obsessive behavior, orthorexia is characterized by nervous fixation on health food, food quality and purity of the diet. Discovered by a doctor of alternative medicine adept, Steven Bratman. The ortoréxico behavior can start innocently, with the desire to cure or prevent chronic disease and improve health. With the aggravation of this behavior, the Orthorexic, in extreme cases, would rather starve than eat the foods considered unclean or unhealthy and thus harmful to your health. The person becomes obsessed and starts to prepare their own food using only known origin of food. It is becoming increasingly compulsive excluding essential items from your diet such as red meat, dairy, sugar and fat without adequate replacement. Its exact etiology and clinical characteristics are not unfamiliar.

**Methods**

As the research population, through research in journal collection sites and scientific articles: Scientific Electronic Library Online - SciELO, PubMed - Public Med; They were selected the maximum number of 47 articles related to the topic orthorexia nervous. Data collection was conducted from January 2014 to February 2016. After a careful process of inclusion and exclusion, we suggest sampling as 17 articles.

**Results**

The data found in the refer approach a subject with little research carried out in academia because such eating behavior has been described for just over ten years. It was possible to observe a higher prevalence of work facing the female common being those that had high economic level, with access to information and good education. Of the 17 articles, an article turned to physical exercise as an adjunct treatment in anxious and depressive disorders, suggesting that it should be prescribed in combination with other therapies in these tables. Another study worked with the clinical case related eating disorder and schizophrenia, and other articles addressed the description and characterization of orthorexia nervous.

**Conclusions**

The influence of media, society and sports means that perfect bodies are synonymous with beauty and success, is affecting men and women to develop eating disorders. This excessive concern with the body and the practice of improper diet is being increasingly adopted by men and women with body image distortion. It is of utmost importance to identify and guide the risk for developing eating disorders, through specialized professionals such as nutritionists, psychologists, physicians and coaches to the success of treatment, to the physical well being and mental of these individuals. Few studies have been conducted to adequately characterize the changes in food consumption of individuals with Orthorexia, making it difficult to approach and nutritional treatment.

## P9 Anthropometric profile and food consumption of players from Basketball Federation of Goiás, Brazil

### Ana Gabriella Pereira Alves, Lana Pacheco Franco, Maria Sebastiana Silva

#### Laboratory of Physiology, Nutrition and Health (LAFINS). Faculty of Physical Education and Dance (FEFD). Federal University of Goiás (UFG). Goiania, Goias, 74690-900, Brazil

##### **Correspondence:** Ana Gabriella Pereira Alves (anagabriela_alves@hotmail.com)

**Background**

The adequate nutritional status prevents injury, reduces fatigue and contributes to better performance. On the other hand, inadequate body composition or food consumption may compromise physical performance, according to the physiological requirements of each sport. In basketball, athletes perform intermittent efforts, with predominance of anaerobic exercise, and occupy a common area for attack and/or defense, which increases the risk of injury. In children and adolescents athletes, the nutritional evaluation is very important because, besides the nutritional demands required for the physical exercise, there is an increase of nutritional requirements for growth and development. In addition, these individuals are typically less informed about adequate nutritional practices and depend on other people for choosing and preparing foods. The aim of this study was to evaluate anthropometric profile and food consumption of players from Basketball Federation of Goiás, Brazil.

**Methods**

In this cross-sectional study, realized in 2013 with 26 children and adolescents, were collected personal (gender and age), anthropometry (weight, height, subscapular and triceps skinfold) and food intake (24-hour recall) data. Were evaluated the body mass index according to age (BMI/age) (WHO, 2007), the body fat percentage (%BF) (SLAUGHTER et al., 1988; DEURENBERG et al., 1990) and the energy and macronutrient intake (carbohydrate, fat and protein) (IOM, 2005). The participants and the legal guardians signed the consent form. The study was approved by the Research Ethics Committee of Federal University of Goiás, Brazil.

**Results**

The study was conducted with 22 men and 4 women, 15.4% were children and 84.6% adolescents, with a mean age of 13.38 (± 3.05) years. Although 53.8% presented both BMI/age and %BF adequate, 34.6% and 11.5% had BMI/age above and below the recommendation, respectively, and 38.4% and 7.7% presented high and low %BF, respectively. The mean dietary energy consumption (DEC) was 1916 (± 626) kcal/day, and 52.9% of DEC was from carbohydrate, 31.0% from fat and 1.4 g/kg body weight from protein (16.1% of DEC). Only 11.54% of the athletes had an adequate DEC, while 73.08% presented insufficient energy intake and 26.92% a DEC above the recommendation. In relation to macronutrients, 76.92% had an adequate intake of carbohydrate, 11.54% a high consumption and 11.54% an intake below the recommendation. From fat, 53.85% showed an adequate intake, 19.23% an insufficient and 26.92% a consumption above the recommendation. Finally, 73.08% of the athletes consumed protein above and 26.92% below the recommendation.

**Conclusions**

A significant number of the participants presented an anthropometric profile and food consumption inadequate for the age group. Thus, they need a nutritional monitoring to maintain appropriate physical conditions for the practice of exercises and sports competition without compromising their growth and development.

## P10 Low efficiency of beta-alanine supplementation for muscle carnosine accumulation in humans

### Pedro Henrique Lopes Perim^1,2^, André Barroso Heibel^3^, Bruno Gualano^1^, Bryan Saunders^1^

#### ^1^Applied Physiology & Nutrition Research Group, University of São Paulo, São Paulo, São Paulo, 05508-900, Brazil; ^2^São Camilo University Centre, São Paulo, São Paulo, 04263-200 Brazil; ^3^Laboratory of Nutritional Biochemistry, University of Brasilia, Brasilia, Federal District, 70910-900, Brazil

##### **Correspondence:** Bryan Saunders, drbryansaunders@outlook.com

**Background**

Chronic supplementation with β-alanine (BA) increases muscle carnosine (MCarn) content, although studies using doses of 3.2 to 6.4 g/day for between 3 and 7 weeks have shown poor efficiency regarding the amount of BA used for MCarn loading despite good whole body retention (>98%; Decombaz et al., 2012). Approximately 2.8% (range: 2.4–5.8%) of BA is used towards MCarn synthesis when assuming that 40% of body mass is muscle mass (Blanquaert et al., 2015). However, these studies based their calculations on percentage changes in MCarn as measured by ^1^H-MRS, while the small muscle groups (*m. deltoid*, *soleus* and *gastrocnemius*) analysed may have underestimated the amount of BA used. Thus, determination of the amount of BA used for MCarn synthesis using chromatographic (*i.e.*, HPLC) quantification of biopsy samples from larger muscle groups is warranted. The purpose was to investigate the effects of 4 weeks of BA supplementation on MCarn content of the *m. vastus lateralis* and determine the estimated amount of BA converted to MCarn.

**Methods**

Twenty-five active males (age 27 ± 5 years, height 1.74 ± 0.09 m, body mass 77.4 ± 11.5 kg) were supplemented with 6.4 g/day of sustained release BA (N = 17) or placebo (PL; N = 8) for 28 days. Pre- and post-supplementation participants provided a muscle biopsy which were subsequently analysed for MCarn content using HPLC. The amount of BA used for carnosine synthesis was calculated by dividing the molar increase in MCarn by the total ingested molar amount of BA and assuming that 40% of body mass is muscle mass. Data were analysed using mixed-models and Pearson’s correlations.

**Results**

MCarn content increased by +11.0 ± 6.7 mmol/kg dry muscle (*P <* 0.0001; Range: +2.42 to +22.10 mmol/kg dry muscle) from baseline in BA, with no change in PL (*P =* 0.99). The estimated amount of BA used was 1.9 ± 1.3% (Range: 0.5 to 4.5%). Pearson’s correlations showed that pre- and post-supplementation MCarn were significantly correlated in the BA group (*P* = 0.01; r^2^ = 0.4), and the increase in MCarn was significantly correlated to the amount of BA used (*P* < 0.0001; r^2^ = 0.9).

**Conclusions**

Four weeks of BA supplementation at 6.4 g/day increased MCarn content in the *m. vastus lateralis* in all individuals, although the estimated amount of ingested BA used was extremely low, confirming previous results using ^1^H-MRS and other muscle groups. Data demonstrate that very little of the BA ingested during supplementation is used for MCarn synthesis and highlights the necessity in further work to optimise BA supplementation in humans.

## P11 Effects of physical exercise in the subjective perception of paresthesia induced by β-alanine supplementation

### Elias de França^1^, Diana Madureira^1^, Yanesko Bella^1^, Fabio Santos Lira^2^ , Jeferson O. Santana^1^, Andre Fukushima^1^, Alex Burton^1^, Erico Chagas Caperuto^1^

#### ^1^GEPAME – São Judas Tadeu University, São Paulo, São Paulo, 03166-000, Brazil; ^2^Exercise and Immunometabolism Research Group, Department of Physical Education, Universidade Estadual Paulista, Presidente Prudente, São Paulo, 19060-900, Brazil

##### **Correspondence:** Erico Chagas Caperuto (ecaperuto@yahoo.com)

**Background**

β-alanine (BA) supplementation has been used to promote increases in muscle carnosine concentration, a pleiotropic molecule (with beneficial effects on health and athletic performance) which can present a side-effect (paresthesia). BA supplementation may promote placebo effect (regarding athletic performance improvement) but, on the other hand (when paresthesia perception is high) it can be uncomfortable. There is no explicit information in the literature whether BA supplementation induced paresthesia is dose-dependent or whether it is affected by physical exercise (PE). The aim of this study was to evaluated paresthesia subjective perception (PSP) induced by different BA supplementation dosages at rest and during PE practice.

**Methods**

In a double blind design 7 healthy males (174 ± 5 cm, 75 ± 6 kg) and 4 women (160 ± 3 cm, 56 ± 4 kg) received (in a crossover and counterbalanced way) two types of treatment (after a 4-hour fasting period): randomly, subjects ingested five different dosages of BA (0.5g, 1.0g, 1.5g, 2.0g and 4 g) or placebo at 1) rest or 2) immediately before PE practice. In 1) participants just sat in a chair for 90 minutes; in 2) participants performed one session of 60 minutes of PE: resistance exercises (bench press and squat: 3 series at 60% 1RM) followed by an aerobic exercise (15 minutes of running at 65% of HRmáx.); participants rested two minutes between resistance exercises series and 5 minutes between exercises. At the end of the PE participants remained at rest for 30 minutes to evaluate the PSP. The PSP was evaluated by a specific questionnaire, intensity of paresthesia sensation and sites of paresthesia perception were evaluated every 10 minutes (for 90 minutes, both at rest and during PE practice).

**Results**

Time to feel the first paresthesia symptoms was different between conditions (rest = 10.8 ± 7.1 minutes; PE = 18.9 ± 19.2, p = 0.03; paired Student's t-test). The PSP intensity induced by BA supplementation was lower in the PE than rest condition in all tested dosages (p = 0.00; two-way ANOVA); the same happened to the body area amount perception, affected by paresthesia (p = 0.00). The PSP intensity showed strong and positive correlation with the BA dosages (rest: *r* = 0.75, p = 0.00; PE: *r* = 0.69, p = 0.00); In the same way, the perception of the number of areas affected by paresthesia sensation also showed positive and moderate correlation with the BA dosages (rest: ρ = 0.45, p = 0.00; PE: ρ = 0.49, p = 0.00). After the PE session PSP was similar to the rest situation (p > 0.05).

**Conclusion**

The PSP intensity as well as the amount of the body sensitive areas with paresthesia symptoms are BA dose-dependent. PE attenuates (in an acute way) the PSP induced by BA supplementation.

## P12 Anthropometric profile and frequency of caffeine consumption by Brazilian recreational runners

### Artur P de Azevedo, Lorruama J Fogaça, Silvia L Santos, João F Mota, Gustavo D Pimentel

#### Clinical and Sports Nutrition Research Laboratory (Labince), Nutrition Faculty (FANUT) – Federal University of Goias (UFG), Goiânia, Goias, 74690-900, Brazil

##### **Correspondence:** Gustavo D Pimentel (gupimentel@yahoo.com.br)

**Background**

The running has become popular around the world and consequently the consumption of ergogenic substances in order to improve performance, although the most widely used is caffeine. In addition, the nutritional counseling is essential for health maintenance, increase athletic performance and control of body composition of athletes. Therefore, the evaluation of anthropometric profile and food behavior can help the professional in best nutritional education runners. The aim was evaluate the anthropometric profile and frequency of caffeine consumption in runners.

**Methods**

A cross-sectional study was performed with convenience sample runners. We evaluated 47 runners (5 and 10 km) of both genders before running. The anthropometric profile performed was the weight, height and handgrip strength. The frequency of caffeine consumption was performed using a standard questionnaire, which were investigated caffeine or caffeine-containing food and supplements. The values were analyzed by median and standard deviation.

**Results**

Most runners were male (n = 28, 59.5%) with mean age of 35.4 ± 7.8 years, BMI of 24 ± 2.4 kg/m^2^ and practitioners of 5 km running (n = 30, 63.8%). The handgrip strength was 40.1 ± 11.3 kg in the right hand and 36.8 ± 9.8 kg in the left hand, indicating normal force for the age group. The handgrip strength was 40.1 ± 11.3 kg in the right hand and 36.8 ± 9.8 kg in the left hand, indicating normal strength for the age group. Regarding to caffeine intake, was observed a greater consumption in morning period (n = 38, 80.8%), followed afternoon (n = 29, 61.7%), evening (n = 11, 23.4%) and midnight (n = 1, 2.1%) with a mean daily frequency of 1.7 times.

**Conclusions**

They are most male runners who training 5 km running. Moreover, caffeine intake is common in the morning period and was not reported consumption close to training, suggesting that these runners do not use food sources of caffeine as an ergogenic aid. In addition, anthropometric data suggest that the active lifestyle influenced beneficially nutritional status once they were classified with normal weight and handgrip strength.

## P13 Effects of a caffeine mouth rinse on dehydration in a 10 km running in highly trained athletes

### Nayra Figueiredo, Marcela Queiroz, Jéssica Santos, João F Mota, Gustavo D Pimentel

#### Clinical and Sports Nutrition Research Laboratory (Labince), Nutrition Faculty (FANUT) – Federal University of Goias (UFG), Goiânia, Goias, 74690-900, Brazil

##### **Correspondence:** Gustavo D Pimentel, gupimentel@yahoo.com.br

**Background**

Caffeine has ergogenic aid important for increasing performance in races. Through the delay fatigue and increase the muscle contraction, it can enhance the ability to perform exercises. Although evidence suggests that caffeine can induce dehydration, effects of mouth rinse with caffeine on dehydration are not investigated. The aim of this study was to analyze if the mouth rinse with caffeine anhydrous interferes in dehydration after a 10 km running.

**Methods**

Double-blind, crossover and placebo controlled study was conducted with 10 adult athletes, 8 men and 2 women with aged of 18 years and highly trained. Subjects were instructed to avoid intense physical exercise and maintaining a balanced diet with caffeine restriction in the two days preceding the test run. On the day run, individuals did mouth rinse with caffeine anhydrous or placebo for 10 seconds and immediately started the 10 km running in a professional running track. The mouth rinse products were separated by a washout period 7d. We measured the baseline and final weight to verify dehydration rate. Student's t test was performed to verify possible statistical difference.

**Results**

We found that the exercise frequency was approximately six times per week and the dehydration rate was 19.01 ± 0.56% in placebo group and 1.49 ± 0.28% in the caffeine group, p = 0.15, no statistical difference.

**Conclusions**

The mouth rinse with caffeine did not alter the dehydration rate when compared to placebo during the 10 km running test in highly trained athletes.

## P14 Caffeine and carbohydrate as strategies nutritional to concurrent strength and high-intensity aerobic exercise: a pilot study

### Daniela S. Inoue^1^, Valéria L.G. Panissa^2^, Paula Alves Monteiro^1,3^, José Gerosa-Neto^1^, Fabrício E. Rossi^1,3^, Erico C. Caperuto^4^, Jason M. Cholewa^5^; Alessandro M. Zagatto^6^, Fábio S. Lira^1^

#### ^1^Exercise and Immunometabolism Research Group, Department of Physical Education, Universidade Estadual Paulista, Presidente Prudente, São Paulo, 19060-900, Brazil; ^2^Department of Sport, School of Physical Education and Sport, University of São Paulo, São Paulo, São Paulo, 05508-900, Brazil; ^3^Center and Prescription Motor Activity Laboratory, Department of Physical Education, Universidade Estadual Paulista, Presidente Prudente, São Paulo, 19060-900, Brazil; ^4^Human Movement Laboratory, Universidade São Judas Tadeu, São Paulo, São Paulo, 03166-000, Brazil; ^5^Department of Kinesiology, Coastal Carolina University, Conway, SC, USA; ^6^Department of Physical Education, of Universidade Estadual Paulista, Bauru, São Paulo, 17033-360, Brazil

##### **Correspondence:** Fabricio E Rossi (rossifabricio@yahoo.com.br)

**Background**

This study investigated the effects of caffeine and carbohydrate intakes on strength performance and the metabolic and inflammatory responses to strength exercise following high-intensity intermittent aerobic exercise.

**Methods**

Seven active males ingested a double-placebo (P), caffeine (CAF; capsule 5 mg.kg^−1^) or carbohydrate (CHO; 20% maltodextrin solution) before performance strength exercise. Participants performed three randomized sessions, consisted a 5000-m high-intensity intermittent aerobic exercise at maximal intensity - V_max_ followed of a strength exercise, performing after the P, CHO and CAF intakes. The blood samples were collected at pre (Pre) and immediately after concurrent strength exercise (Post).

**Results**

Similar response on number of repetitions and total volume performed in all conditions. There was a main effect of time for glucose, lactate and IL-6 (*p* < 0.05). When compared the changes between condition (post minus pre-values), there was lower glucose in CAF in relation to the CHO (CAF = 5.0 ± 10.4 vs CHO = 27.8 ± 20 vs P = 15.1 ± 14, *p* = 0.039) and higher IL-6 levels (CAF = 11.9 ± 9.2 vs CHO = -2.4 ± 1.7 vs P = 4.3 ± 11.7, *p* = 0.022). There was statistically significant interaction for glucose, lactate (*p* < 0.001).

**Conclusions**

CAF and CHO intakes did not improve strength performance during concurrent strength training in active males, however, caffeine can affect the immunometabolic response.

## P15 Is there a need of protein ingestion before sleep to maximize muscle hypertrophy? – a systematic review of recent data

### Laís Monteiro Rodrigues Loureiro, Caio Eduardo Gonçalves Reis

#### Laboratory of Nutritional Biochemistry, University of Brasilia, Brasilia, Federal District, 70910-900, Brazil

##### **Correspondence:** Lais Monteiro Rodrigues Loureiro, laismonteirorp@hotmail.com

**Background**

Increases in skeletal muscle mass can be achieved through resistance exercise training and adequate diet. The recommendation of protein intake for maximize muscle protein synthesis involves 1.5–2.2 g/kg.day^−1^ or 0.2–0.30 g/kg per meal of high-quality protein source. Protein timing is a dietary strategy designed to optimize the net muscle protein accretion. Timing of protein ingestion in relation to overnight recovery is a topic of recent investigations. Pre-sleep feeding is a time when protein intake may provide a marked benefit to remodel muscle proteins. Thus, the aim of this study is to analyse the clinical trials that evaluated the effects of pre-sleep protein consumption on muscle protein synthesis during overnight recovery.

**Methods**

A systematic literature search was conducted on PubMed database (English, Spanish and Portuguese) seeking articles published until September 2016 using a combination of the following keywords: ‘sleep’, ‘overnight’, ‘muscle’, and ‘protein’. Clinical trials with human subjects which analyzed the effects of pre-sleep protein intake on overnight protein synthesis were included. Studies characteristics and muscle protein synthesis parameters (‘whole-body protein synthesis’ and ‘mixed-muscle protein fractional synthetic rate’) were appraised.

**Results**

Eighteen studies were identified through database searching. After eligibility assessment, four studies were selected for the final analysis. Compared with placebo treatment (water), 30 or 40g of protein (casein) intake prior to sleep seems to improve the whole-body protein synthesis rates in healthy adult men during overnight period (7.5 to 9.0 hours) with or without resistance exercise training in the evening before. The studies found no effect of before sleep protein intake on mixed-muscle protein fractional synthetic rate.

**Conclusions**

The present study is the first systematic review that examines the effects of pre-sleep protein supplementation on muscle protein synthesis rate during overnight period. Overall, these findings suggest that pre-sleep protein intake has benefit effects on overnight whole-body protein synthesis rates. It remains to be established whether ingestion of a moderate amount of protein increases overnight muscle protein synthesis parameters in all populations. Therefore, more high-quality clinical trials are required to confirm these results.

